# Phytate and Butyrate Differently Influence the Proliferation, Apoptosis and Survival Pathways in Human Cancer and Healthy Colonocytes

**DOI:** 10.3390/nu13061887

**Published:** 2021-05-31

**Authors:** Lidia Hanna Markiewicz, Anna Maria Ogrodowczyk, Wiesław Wiczkowski, Barbara Wróblewska

**Affiliations:** 1 Department of Immunology and Food Microbiology, Institute of Animal Reproduction and Food Research, Polish Academy of Sciences, Tuwima 10, 10-748 Olsztyn, Poland; a.ogrodowczyk@pan.olsztyn.pl (A.M.O.); b.wroblewska@pan.olsztyn.pl (B.W.); 2 Department of Chemistry and Biodynamics of Food, Institute of Animal Reproduction and Food Research, Polish Academy of Sciences, Tuwima 10, 10-748 Olsztyn, Poland; w.wiczkowski@pan.olsztyn.pl

**Keywords:** cancer colonocytes, healthy colonocytes, phytate, butyrate, apoptosis, cell cycle, cell survival, diet

## Abstract

The colonic epithelium is never exposed to a single factor, therefore studies on the effect of combinations of factors naturally and persistently present in the intestines are of special importance for understanding the phenomena occurring at this place. The aim of the study was to investigate the combined effect of 1 mM phytate and 1 mM butyrate (PA1B1) on cell lines derived from cancer (HCT116 and HT-29) and healthy (NCM460D) human colonic epithelium. Colorimetric and flow cytometry methods were used to determine the proliferation rate, cell cycle, and apoptosis. Selected markers of proliferation, inflammatory, and survival pathways were investigated at the mRNA and/or protein level. The combination of phytate and butyrate disturbed the cell cycle and triggered apoptosis and/or death in both studied cancer colonocytes to a higher extent compared to healthy colonocytes. Moreover, in healthy colonocytes, phytate activated the survival pathway without stimulation of inflammatory response. This may indicate that the response of healthy colonocytes to phytate protects colonic epithelium from the loss of integrity and tightness that would occur if inflammation developed. Based on the obtained results we postulate that studies on both cancer and/or healthy colonocytes should be carried out in the presence of butyrate as the permanent component of colonic contents. This should be of special importance when anti-proliferative/pro-apoptotic activity or inflammatory status of colonocytes is to be investigated.

## 1. Introduction

The physiological status of intestinal epithelium is an effect of multiple factors among which diet and intestinal microbiota are the most important ones. Recent reports on the health consequences of particular diet components and nutritional lifestyle shed light on the mutual dependencies of intestinal microbiota and its metabolites, diet, and intestinal epithelium [1,2]. More and more attention is being paid to the dietary plant-derived compounds and their protective effect against civilization diseases at the systemic and cellular level.

A well-balanced diet is rich in cereals (whole grain), legumes (soy, lentils, peas), and nuts, which are a source of fibers and phytates, among others. Fibers are non-digestible carbohydrates that are fermented by colonic bacteria and stimulate microbial production of short chain fatty acids (SCFAs) [3]. Among SCFAs, butyrate, propionate, and acetate constitute about 90% of the pool, and butyrate is the key bacterial metabolite that, at an appropriate concentration, ensures proper functioning of the intestinal epithelium in the aspect of gut barrier, epithelium renewal, immune response, and ion absorption [4,5].

Butyrate at physiological concentrations exerts a pro-apoptotic effect on cancer colonocytes by an epigenetic mechanism involving histone acetylation and apoptosis induction. The most commonly tested concentrations of butyrate range from 2 to 5 mM and are physiologically relevant [6,7,8], although cells located in the colonic crypts are exposed to lower butyrate concentrations (about 0.5–1 mM). Moreover, the metabolic capacity of epithelial cells is limited to 1–2 mM of butyrate. [9]. Above this concentration butyrate acts as acetyl-CoA donor increasing histone acetylation and as a direct inhibitor of a histone deacetylase (HDA) which leads to changes in epigenetic regulation of genes including those involved in cell cycle and apoptosis control. A stimulating effect of low (0.5 mM) doses of butyrate on cancer cell proliferation and the same effect of higher (above 2 mM) levels of butyrate on healthy colonocytes has been explained by the Warburg effect [10]. In general, it relies on utilization of butyrate by healthy cells as a main energy source which avoids intracellular accumulation of butyrate and its pro-apoptotic effect. In cancer cells, which have their energy metabolism switched from butyrate to glucose, butyrate accumulates leading to the changes of epigenetic regulation.

Phytates (salts of phytic acid, PA, inositol hexakisphosphate, *Ins*P_6_, IP6) are present in grains, legumes, seeds, and nuts, and are considered as antinutrients since bind metal ions (calcium, zinc, iron, copper) and proteins lowering their bioavailability and digestibility [11]. During passage through the human gastrointestinal tract, 80–90% of phytate is hydrolyzed mainly by microbial phytases and this activity is higher in vegetarians’ than in omnivores’ microbiota [12,13]. The daily dietary phytate levels vary depending on the type of diet and country, although some trends can be found for Western-type diets containing low (200–350 mg/d) levels of phytate and for vegetarian diets (over 1000 mg/d) rich in cereals and legumes. Besides its well-known undesirable properties, PA has shown its beneficial effects on cancer cells by activating apoptotic machinery as well as by suppressing cell survival pathways [14,15,16]. The anti-tumorigenic effect of phytate has been widely investigated. However studies focused mainly on its in vitro action on cancer cell lines (derived from colon, prostate, breast and lung cancers) and on animals in rodent models of cancer and on xenograft models [17,18]. Phytic acid acts on cancer cells in a time and dose dependent manner and its effect depends on the type of cells tested [19,20].

Both phytate, as a diet compound, and butyrate, as an intestinal microbiota metabolite, are constituents of colon contents and their concentrations vary and are strictly dependent on diet and metabolic activity of gut microbiota. What is important, in the latest comprehensive scientific review, diet components such as whole grain and total fiber (source of phytates and substrate for butyrate production, respectively) are indicated as “probable protective factors” against colorectal cancer [21]. Up to now, the effect of phytate and butyrate on colonocytes was investigated separately. In this study we characterize the simultaneous effect of phytate (a dietary compound) and butyrate (bacterial metabolite of dietary fibers) on human colonocytes derived from cancer (HCT116 and HT-29 cell lines) and normal tissue (NCM460D cell line). We have chosen cells of different origin since the evidence exists that tumor cells derived from different progenitor cell types are characterized by different signal transduction patterns, therefore their response to anti-tumor agents that disrupt these patterns may be different [22,23]. The aim of the study was to investigate the cell cycle, proliferation, and apoptotic processes in human colonocytes in the presence of both of the agents. Moreover, we investigated markers of pathways known to be involved in the regulation of cell inflammatory responses and survival.

## 2. Materials and Methods

### 2.1. Phytate and Butyrate

Phytic acid from rice (PA; in a form of sodium salt; P8810, Sigma Poznań, Poland) and sodium butyrate (B5887, Sigma) were used in the studies. Stocks of phytic acid (200 mM and 400 mM) were prepared in PBS, filter sterilized (0.22 µm), aliquoted, and stored at 4 °C for no longer than a week. Stocks of butyrate (1 M) were prepared in PBS, aliquoted and stored in cryovials at −20 °C.

### 2.2. Characteristics of Phytate Preparation

The composition of the tested phytic acid preparation was analyzed using the HPLC system (LC-20, Shimadzu, Japan) coupled with a QTRAP 5500 mass spectrometer (AB SCIEX, Toronto, ON, Canada). Briefly, the chromatographic determinations of phytate dissolved in ultrapure water were performed on the Luna C18 column (250 × 2 mm 5 µm, Phenomenex, Torrance, CA, USA) at 40 °C with a flow rate of 0.2 mL/min. The elution was conducted using a solvent gradient system consisting of solvent A (5% acetonitrile aqueous solution with 5 mM ammonium acetate and 5 mM dihexylammonium acetate) and solvent B (100% acetonitrile). Compounds were identified based on the comparison of their retention time and the presence of the respective parent and daughter ion (negative) pairs (IP6: 659–561 *m*/*z*, IP5: 579–481 *m*/*z*, IP4: 499–401 *m*/*z*, IP3: 419–321 *m*/*z*, IP2: 339–241 *m*/*z*, IP1: 259–79 *m*/*z*, *myo*-inositol: 179–161 *m*/*z*) with the authentic standards. The applied standards are listed in Table S1.

### 2.3. Cell Lines and Growth Conditions

Two cell lines derived from colon cancer tissue: HT29 (Sigma) and HCT116 (American Type Culture Collection (ATCC); LGC Standards, Łomianki, Poland) and one cell line derived from normal (non-tumorigenic) colon tissue, namely NCM460D (under a license from InCell Corp., San Antonio, TX, USA) were used in the study. HT29 and HCT-116 were routinely cultured in McCoy5A medium (Sigma). NCM460D cells were cultured in M3A medium containing antibiotics (InCell). Media applied for culturing of cancer and healthy colonocytes were different but optimal for each type of the cells to maintain cell line-specific growth conditions and ensure that observed cell response is solely due to the factors tested. All media were supplemented with 10% non-inactivated fetal bovine serum (FBS, Gibco) and McCoy5A medium, and with antibiotics (100 U/mL penicillin, 100 µg/mL streptomycin, and 25 µg/mL amphotericin B). Cells were incubated at 37 °C, in an atmosphere of 5% CO_2_ and 90% humidity. The media were changed every two (HT29 and HCT-116) or three (NCM460D) days, according to the provider’s recommendation. After reaching 70–80% confluency, the cells were subcultured by trypsinization (0.05% trypsin-EDTA, Thermo Fisher, Life Technologies) and split in a 1:6 (HCT-116 and HT-29 cells) or 1:3 (line NCM460D) ratio and cultured in fresh medium. Cells at passages 12–27 (NCM460D) and 21–38 (HCT11 and HT-29) were used.

### 2.4. Experimental Design

The cells grown as described in Section 2.3 were harvested by trypsinization and counted using a cell counter (Scepter, Merck Millipore). For proliferation tests, the cells were seeded into 96-well plates at a density of 1 × 10^4^ cells/well (HCT-116 and HT-29) or 4 × 10^4^ cells/well (NCM460D). For flow cytometry analyses and RNA extraction, the cells were seeded into 12-well plates at a density of 5 × 10^5^ per well. For protein isolation, the cells were seeded at a concentration of 1 × 10^6^ cells/well in 6-well plates. The cells were subjected to experiment 24 h after seeding. After that time, the medium was replaced with a fresh medium containing phytate (PA) and/or butyrate and incubated for the next 24 h. In proliferation tests, the range of applied concentrations was the widest: 0.5 to 5.0 mM for phytate and from 0.2 to 50 mM for butyrate. For all other analyses, the concentration of 1 mM of phytate (PA1.0) and 1 mM of butyrate (B1.0) was chosen and they were tested separately and in combination (PA1B1). The total volume of PA and butyrate added to medium did not exceed 5%. Medium without PA and butyrate served as a control (CTRL) and medium supplemented with PBS (at the volume equal to the total PA and butyrate volumes) was tested to determine the effect of the vehicle (VEH).

### 2.5. Proliferation Test

After 24 or 48 h incubation with PA and/or BUT, 10 µL of Cell Proliferation Reagent (Wst-1, cat. no 11644807001, Roche) was added to each well containing 100 µL of medium and incubated for 2 h (HCT116 and HT-29) or 4 h (NCM460D). The time of incubation was chosen based on pre-tests carried out as recommended by the manufacturer. After that time absorbance at 450 nm was read (spectrophotometer; ASYS UVM 340). Impact of tested factors on cell proliferation was expressed as the proliferation rate (PR%) and was calculated according to the formula: (Asample − Ablank)/(Acontrol − Ablank) × 100; where A—absorbance at 450 nm. Influence of tested factors on %PR was analyzed against non-treated control cells (CTRL).

### 2.6. Apoptosis and Cell Cycle Determination with Flow Cytometry

The determination of the external exposition of phosphatidylserine (PS), as an apoptotic marker, was carried out using a FITC Annexin V/Dead Cell Apoptosis kit (V13242, Invitrogen) according to the manufacturer’s instructions including FMO (fluorescence minus one) control. Briefly, cells after completion of the experiment were washed with PBS without calcium and magnesium ions, trypsinized, transferred to 2.0 mL test tubes, and washed again with PBS. After the washing step, the cells were centrifuged and the cell pellet was resuspended to the concentration of 1 × 10^6^ cells/mL in annexin-binding buffer. Staining of cells was carried out with anticoagulant, Annexin V conjugated with FITC (5 μL of component A per 100 μL of cell suspension) and/or propidium iodide (1 μL of the 100 μg/mL solution/100 μL of cell suspension). After incubation for 15 min at room temperature, 400 μL of annexin-binding buffer was added and fluorescent intensities of FITC (λexc = 494 nm and λem = 518 nm) and PI (λexc = 535 nm and λem = 617 nm) was measured by flow cytometry. Hydrogen peroxide (10%) was used as an inducing agent for obtaining control cells showing necrotic cell death. Three cellular subpopulations were evaluated: viable cells (Annexin V−/PI−), early apoptotic cells (Annexin V+/PI−, EarlyAp), and late apoptotic and necrotic/damaged cells (Annexin V+/−/PI+, LateAp/Dead) [24,25].

For the confirmation of appearance of subdiploid-fragmented nuclei derived from late apoptotic cells and necrotic cells (the SUB G1 subpopulation), the cell cycle analysis previously described by the authors of [26,27] was performed. Briefly, cells after completion of the experiment were harvested, washed with PBS, and resuspended in 1 mL of lysis buffer (1 mg/mL trisodium citrate, 0.1% Triton X-100, 0.05 mg/mL propidium iodide and 1 mg/mL RNase A (P4875 Sigma)). After incubation (4 h, 4 °C), the released nuclei were resuspended by agitation with a Pasteur pipette. The fluorescence intensity (exc = 536 nm, em = 617 nm) was measured by flow cytometry. Cytometric analyses were carried out in triplicate with the use of a BD LSR Fortessa Cell Analyzer (USA) and BD FACS Diva™ software. At least 20,000 events per sample were analyzed.

### 2.7. RNA Isolation and Reverse Transcription

Cells grown in 12-well plates were washed with cold PBS, then covered with RNAlater (500 µL; Sigma) and incubated overnight at 4 °C. Next, the cells were transferred to an Eppendorf tube and stored at −20 °C. Cells were washed in PBS to remove RNAlater and after centrifugation (1000× *g*, 2 min) the cell pellet was subjected for total RNA extraction using a protocol provided with Universal RNA Isolation Kit (Eurx, Gdańsk, Poland). Concentration and purity of RNA were determined spectrophotometrically (NanoDrop). Reverse transcription of 250 µg of total RNA was carried out in a total volume of 20 µL with the use of U of *AMV* reverse transcriptase (Eurx) and 10 µM of random hexamers (Eurx) applying a protocol recommended by the producer that included incubation at 50 °C for 50 min.

### 2.8. Gene Expression Analysis

Real-time amplifications were performed in QuantStudio 6 Flex system (Thermo Fisher, Life Technologies, Warsaw, Poland). One microliter of two-fold diluted cDNA was used in the reaction mixture containing 10 µL of 2× Power SYBR Green PCR Master Mix (Thermo Fisher Scientific), 0.8 µL of each primer (10 µM) and water filled up to 20 µL. The list of primers used in this study is shown in Table S2. Data were analyzed in Thermo Fisher Scientific Cloud using a Relative Quantity app applying a ΔΔCt method, using *ACTB* (β-actin) as a reference gene.

### 2.9. Protein Extraction and Immunoblotting

After completion of the experiments, plates were placed on ice and the cells were washed twice in cold PBS and cryopreserved (−70 °C) in 500 µL of RIPA buffer with inhibitors (SIGMA*FAST* Protease Inhibitor Tablets and Phosphatase Inhibitor Cocktail 2 (S8820 and P5726, respectively) from Sigma).

Protein concentration was assessed with the use of Direct Detect™ Assay-free Cards (EMD Millipore) and a Direct Detect Spectrometer (EMD Millipore). Ten or twenty micrograms of proteins were loaded onto 12.5% polyacrylamide gel in reducing conditions and separated in Tris-glycine buffer (pH 8.3) as described previously [28]. Proteins were transferred onto a Immun-Blot^®^ Low Fluorescence PVDF membrane (Bio-Rad) and probed with primary antibodies: anti-NFκB p65 (RelA), anti-pro-caspase 3, anti-active caspase 3, anti-ERK1+ERK2, anti-ERK1(phospho T202) + ERK2(phospho T185), and anti-β-actin, from Abcam (Cambridge, UK). Primary antibodies: anti-Akt1 and anti-p-Akt1 (Thr 308) were purchased from Santa Cruz Biotechnology (Dallas, TX, USA). Secondary antibodies labeled with fluorescent dyes: donkey anti-goat (Alexa Fluor 680), goat anti-mouse IgG (Alexa Fluor Plus 680), and goat anti-rabbit (Alexa Fluor Plus 800) were from Thermo Fisher Scientific (Life Technologies, Poland). Membranes were scanned in the ChemiDoc Imaging System (Bio-Rad, Warsaw, Poland) and analyzed in Image Lab software (Bio-Rad). Levels of all investigated proteins were normalized to β-actin. The changes in the phosphorylation level of Akt and ERK1/2 were presented as the ratio of phosphorylated to non-phosphorylated protein (both were first normalized to β-actin).

### 2.10. Statistical Analysis

Data are expressed as mean ± SD from three independent experiments repeated in duplicate or triplicate samples. Results of proliferation tests were analyzed with Student’s *t*-test. Data obtained from cytometric, Western blot, and gene expression analyses that met requirements for parametric tests were analyzed with one-way ANOVA followed by post hoc Duncan test. All other data were analyzed with a nonparametric Kruskall–Walis test. All calculations were performed with Statistica v. 12 (Statsoft, Kraków, Poland). Differences were considered significant at *p* ≤ 0.05.

## 3. Results

### 3.1. The Composition of Phytate Preparation

Analysis of the tested phytate preparation showed that it was comprised mainly of hexa- and penta-inositol phosphates that constituted 62 and 36% of total inositol phosphates, respectively (Table 1). Mono-, di-, tri-, and tetra inositol phosphates accounted for only about 2% of total inositol phosphates.

### 3.2. Cell Proliferation

**Figure 1:**
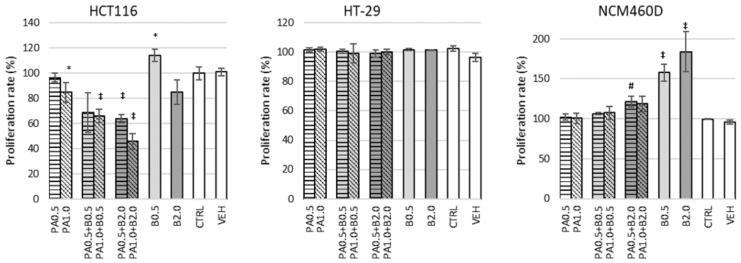
Proliferation rate (%) of HCT116, HT-29, and NCM460D cells in the presence of butyrate or phytate applied separately or in combination. White bars—control cells incubated without phytate or butyrate (CTRL) or with PBS as a vehicle (VEH). Bar patterns: horizontal and diagonal—cells incubated with phytate at a concentration of 0.5 mM (PA0.5) or 1.0 mM (PA1.0), respectively. Light-gray and dark-gray bars—cells incubated with butyrate at a concentration of 0.5 mM (B0.5) or 2.0 mM (B2.0), respectively. Incubations were carried out at 37 °C for 24 h. Values significantly different from CTRL at *p* < 0.05, *p* < 0.01, and *p* < 0.001 are depicted with *, #, and ‡, respectively.

No effect of butyrate was observed on the proliferation rate of cancerous HCT116 cells after 24 h of incubation (Figure 1 and Figure S1A). In the 48 h cultures, the lowest butyrate concentrations (0.2 and 0.5 mM) stimulated (*p* ≤ 0.001 and *p* ≤ 0.05, respectively), whereas concentrations of 2 mM and higher significantly reduced (*p* ≤ 0.001) proliferation of these cells (Figure S1A). Doses of 0.5 and 1.0 mM of phytate had no effect on cell proliferation of HCT116, whereas higher (2–5 mM) reduced it up to 25% (*p* ≤ 0.001; Figure S1A) after 24 h of incubation. A mixture of 1.0 mM of phytate and 2 mM of butyrate showed the strongest inhibition of cell proliferation up to 50% of the control after 24 h (*p* ≤ 0.001; Figure 1).

HT-29 cells were more resistant to butyrate action than HCT116 (Figure 1 and Figure S1B). Only high concentrations of butyrate (above 10 and 5 mM for 24 h and 48 h cultures, respectively) reduced the proliferation rate up to 100% (50 mM of butyrate, *p* ≤ 0.05 and *p* ≤ 0.001; Figure S1B). Phytate applied alone for 24 h had no effect on the proliferation rate of HT-29 cells (Figure S1B) as did all tested combinations of PA and butyrate (Figure 1).

The NCM460D cell line responded to butyrate in a different way than tumorigenic cells. Concentrations from 0.2 to 2.0 mM stimulated proliferation after 24 h (*p* ≤ 0.05 and *p* ≤ 0.001), whereas after 48 h only 0.5 mM concentration of butyrate showed that effect (*p* ≤ 0.05; Figure S1C). Phytate did not change the proliferation rate of NCM460 cells at tested concentrations (Figure S1C); however, it diminished the stimulating effect of low doses (0.5 mM) of butyrate (Figure 1).

Based on the obtained results (a cell line-specific response to tested compounds) and literature data [10], a concentration of 1 mM of butyrate (B1.0) and 1 mM of phytate (PA1.0) were chosen, as the minimal concentrations that can have an observable effect on colonocytes. A combination of these factors at selected concentrations (PA1B1) was investigated for their synergistic effect.

### 3.3. Cell Cycle Analysis and Apoptosis

**Figure 2:**
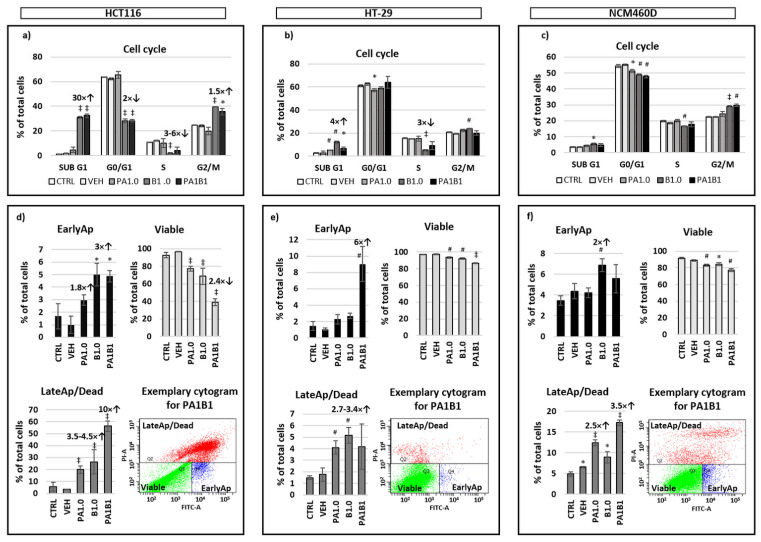
Results of flow cytometry analyses of HCT116, HT-29, and NCM460D cell lines. (**a**–**c**) cell cycle analysis of cells stained with propidium iodide. Phases of the cell cycle: SUB G1, G0/G1—cells at the stage of Gap 1 and Gap 0; S—synthesis of DNA; G2/M—cells in the phase of Gap 2 and mitosis. (**d**–**f**) analysis of apoptosis based on the presence of annexin V. EarlyAp—cells the at early apoptosis stage; LateAp/Dead—cells at the late apoptosis stage and dead ones. CTRL—control cells cultured without supplements; VEH—vehicle, cells cultured in medium with PBS (phytate and butyrate diluent); PA1.0—cells incubated in medium containing 1 mM phytate; B1.0—cells incubated in medium containing 1 mM butyrate; PA1B1—cell incubated in medium with both PA1.0 and B1.0. Values that differ significantly from CTRL at *p* < 0.05, *p* < 0.01, and *p* < 0.001 are marked with *, #, and ‡ respectively. Fold changes over the columns depict values different from CTRL, up or down arrows indicate an increase or a decrease from CTRL.

In HCT116 cells, butyrate had the prevailing effect on the cell cycle by a significant increase (*p* ≤ 0.001) of proportions of apoptotic/necrotic cells (SUB G1 phase) and those undergoing G2 arrest (G2/M phase) and decreasing cells in G0/G1 and in the S phase (Figure 2a). Analysis with annexin V showed significantly higher proportions of early apoptotic cells in B1.0 and PA1B1 samples (*p* ≤ 0.05; Figure 2d). Proportions of late apoptotic/necrotic cells were also higher (*p* ≤ 0.001) in PA1.0, B1.0, and PA1B1 samples with the biggest changes (10-fold higher) in the latter ones. As a consequence, the number of viable cells in PA1.0, B1.0, and PA1B1 samples were reduced compared to the control (*p* ≤ 0.001).

In HT-29 cells, butyrate significantly increased the size of subpopulation at SUB G1 and G2/M phase (*p* ≤ 0.01) and decreased that in the S phase (*p* ≤ 0.001; Figure 2b). The increase of proportions of SUB G1 cells in the presence of phytate alone and with butyrate was also significant (*p* ≤ 0.01) although less profound. Analysis with annexin V showed that HT-29 cells treated with PA1B1 had the highest proportion of early apoptotic cells (*p* ≤ 0.01; Figure 2e). All treatments significantly reduced the number of HT-29 viable cells, and increased proportions of late apoptotic and necrotic ones; however, in the latter case the significant changes were observed only for B1.0 and PA1.0 samples (*p* ≤ 0.01; Figure 2e).

In NCM460D cells, only butyrate (B1.0) significantly increased the cell percentage at SUB G1 phase and lowered those at S phase (*p* ≤ 0.05; Figure 2c). Phytate alone or with butyrate had no impact on the number of SUB G1 and S phase cells (*p* ≤ 0.01). All investigated factors decreased the percentage of cells at the G0/G1 phase, whereas only butyrate alone (B1.0) and with phytate (PA1B1) significantly increased proportions of cells at G2/M (*p* ≤ 0.001 and *p* ≤ 0.01, respectively). Analysis of annexin V confirmed that butyrate (B1.0) increased proportions of early apoptotic cells (*p* ≤ 0.01), whereas phytate alone and in combination with butyrate increased the population of late apoptotic/dead cells and decreased that of viable cells (*p* ≤ 0.05; Figure 2f).

### 3.4. Analysis of Gene and Protein Expression

#### 3.4.1. Basal mRNA Expression in Untreated Cells—Comparison of Cell Lines

Relative basal expression of examined genes in non-treated cells was carried out in reference to NCM460 cells (Figure S2). Analysis of genes involved in regulation of apoptosis (*BCL2, BCLXL*), immune response (*NFkB1*, *IL8*, *TNFα*), and cell proliferation (*PTEN*, *iNOS*) revealed significant differences in their expression in the examined cell lines. HCT116 cells had the highest expression of anti-apoptotic *BCL2* and *TNFα* and the lowest level of pro-apoptotic *PTEN*. The HT-29 cells expressed the highest anti-apoptotic *BCLXL*, as well as immunologically relevant *IL8* and *NFkB1*, whereas NCM460D stood out with the highest *iNOS* expression (Figure S2).

#### 3.4.2. Effect of Phytate and/or Butyrate on Gene and Protein Expression in Treated Colonocytes

**Figure 3:**
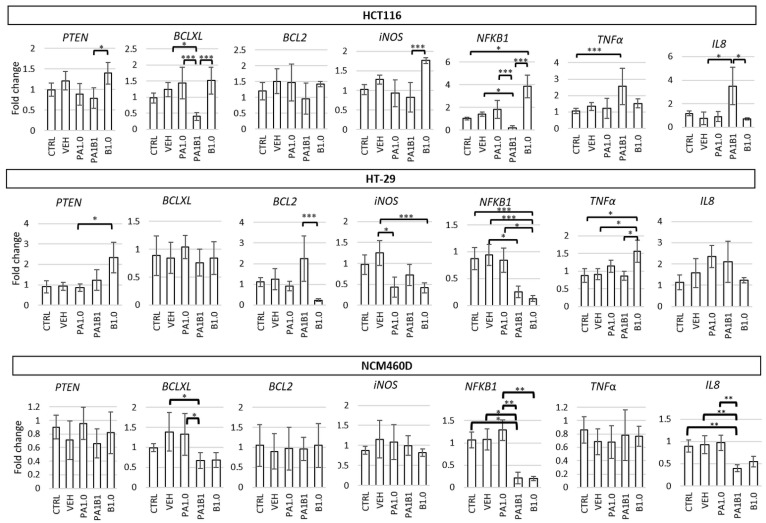
Impact of 1 mM butyrate (B1.0), 1 mM phytate (PA1.0) and their combination (PA1B1) on the expression of *PTEN* (phosphatase and tensin homolog), *BCLXL* (B-cell lymphoma—long isoform (Bcl-xL))*, BCL2* (B-cell lymphoma protein 2), *iNOS* (inducible nitric oxide synthase), *NFΚB1* (nuclear factor kappa B p105 subunit), *TNFα* (tumor necrosis factor alpha), and *IL8* (interleukin 8) genes in HT116, HT-29, and NCM460D cells. The values are significantly different at *p* < 0.05, *p* < 0.01, and *p* < 0.001 are marked with *, **, and ***, respectively.

**Figure 4:**
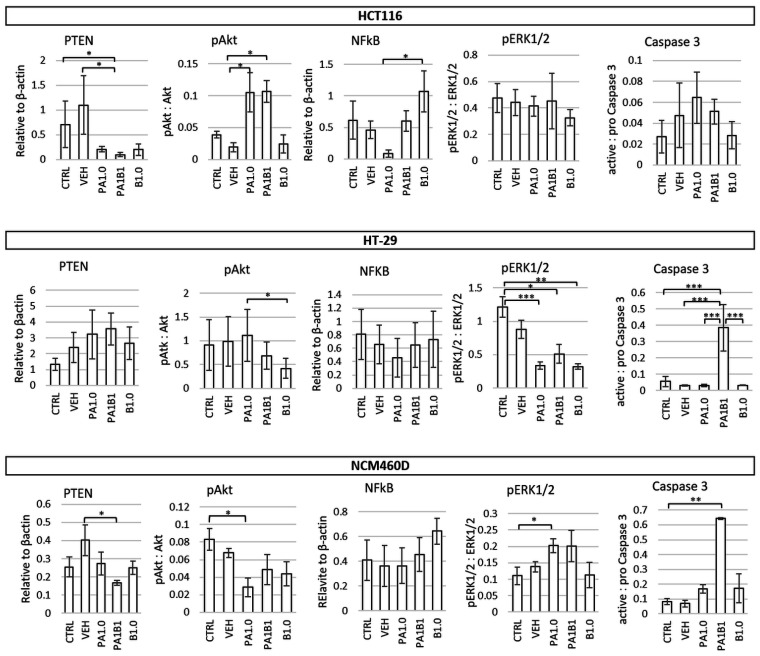
Impact of 1 mM of phytate (PA1.0) and 1 mM of butyrate (B1.0) applied alone or in combination (PA1B1) on the level of cellular proteins or their phosphorylation. PTEN (phosphatase and tensin homolog), pAkt (ratio of phosphorylated to unphosphorylated form of Akt), NFκB (nuclear factor kappa B p65), pERK1/2 (ratio of phosphorylated to unphosphorylated form of ERK1/2), active caspase 3 (ratio of active form of caspase 3 to pro-caspase 3) in HT116, HT-29, and NCM460D cells. The values are significantly different at *p* < 0.05, *p* < 0.01, and *p* < 0.001 are marked with *, ** and ***, respectively.

In HCT116 cells the most significant alterations in gene expression were observed in PA1B1-treated cells in which *BCLXL* and *NFκB1* showed the lowest (*p* ≤ 0.001 vs. PA1.0 and B1.0) and *TNFα* and *IL8* the highest expression (*p* ≤ 0.05 vs. CTRL and VEH, respectively; Figure 3). Butyrate alone significantly increased the expression of *NFκB1* compared to control (*p* ≤ 0.05) as it also did for *PTEN* and *iNOS* when compared to PA1B1 treatment (*p* ≤ 0.05 and *p* ≤ 0.001, respectively). Phytate alone showed no significant impact on the expression of examined genes when compared to the control cells. Analysis of protein expression showed lowered levels of PTEN (an inhibitor of the Akt pathway) in all treated cells and elevated phosphorylated Akt (pAkt) in PA1.0 and PA1B1 treated cells (*p* ≤ 0.05; Figure 4). Reduced levels of cyclin A in B1.0 and PA1B1 cells (*p* ≤ 0.05 and *p* ≤ 0.001, respectively; Figure S3) confirmed the inhibition of DNA replication and disturbances in cell cycle progression.

HT-29 cells treated with phytate or butyrate had lowered expression of *iNOS*, and the butyrate-treated cells (B1.0 and PA1B1) had also lowered *NFκB1* expression compared to control cells (*p* ≤ 0.001, Figure 3). Moreover, in cells treated only with butyrate, the expression of pro-apoptotic *PTEN* and anti-tumor *TNFα* increased (*p* ≤ 0.05), whereas anti-apoptotic *BCL2* was reduced (*p* ≤ 0.001) compared to PA1B1 treatment. Expression of *BCLXL* and *IL8* was not affected by the tested factors (Figure 3). A significant increase of active caspase 3 levels confirmed apoptosis in PA1B1-treated HT-29 cells (*p* ≤ 0.001 vs. control and other treatments, Figure 4). The observed trend for an increase of PTEN and a decrease of its downstream target (phosphorylated Akt) indicated on the induction apoptosis through the PTEN-PI3K-Akt pathway in butyrate-treated HT-29 cells and confirmed the flow cytometry results regarding apoptosis (Figure 2b). Lowered level of cyclin A in butyrate-treated cells (B1.0 and PA1B1; *p* ≤ 0.05; Figure S3) confirmed the inhibition of DNA synthesis observed in cytometric analyses (Figure 2b). The level of pERK1/2 decreased in all treated cells (*p* ≤ 0.05; Figure 4).

In NCM460D cells the expression of *PTEN*, *iNOS*, *BCL2*, and *TNFα* at mRNA level was not affected by phytate and butyrate. *BCL-XL*, *NFΚB1*, and *IL8* were significantly reduced in PA1B1 treated cells (*p* ≤ 0.05, *p* ≤ 0.05, and *p* ≤ 0.01, respectively, Figure 3), whereas the two latter genes were also reduced in B1.0-treated cells (*p* ≤ 0.01, Figure 3). At protein level, NFκB expression was not changed in all samples, PTEN was significantly lowered in PA1B1-treated cells, whereas pAkt showed a tendency to decrease in all treated cells (a statistical significance was confirmed only for PA1.0-treated cells (*p* ≤ 0.05, Figure 4). On the other hand, the activated extracellular signal-regulated kinase (phosphorylated ERK1/2) increased in PA1.0 and PA1B1 treated cells. However the statistical significance (*p* ≤ 0.05) was confirmed only for PA1.0-treated cells. Only in PA1B1-treated cells the level of active caspase 3 increased and was over seven times higher compared to control cells (*p* ≤ 0.01; Figure 4) indicating the ongoing apoptotic process. The level of cyclin A was slightly lower (Figure S3) in all treated cells as compared to controls.

The effects of phytate and/or butyrate on cellular processes are summarized in Table 2.

## 4. Discussion

Food components act on human organisms directly providing nutrients and indirectly through modulation of the composition and metabolic activity of intestinal microbiota. It has been reported that both bacteria and food components impact the physiological and immune status of cells lining the intestine [29,30]. Phytate, due to inactivation of indigenous plant phytases during food processing, undergoes hydrolysis as an effect of microbial activity in the human gastrointestinal tract. The hydrolysis is, however, not complete; therefore phytate can still affect intestinal epithelium [11]. The phytate preparation investigated in this study consisted of 67% InsP6 and 33% InsP5, therefore its effect on colonocytes may be attributed to both of these inositol phosphates.

In this study two cell lines derived from colorectal cancer (HCT116 and HT-29) and one derived from normal colonic epithelium (NCM460D) were used as models of cancer and healthy colonocytes, respectively. The NCM460D line, despite the fact that acquired some karyotype traits found in cancer cells (communication from InCell Corp.), is used as a healthy reference in studies on mechanisms of carcinogenesis and anticancer activity [31,32,33,34,35]. In our study, NCM460D cells responded to butyrate in a manner characteristic for healthy colonocytes, i.e., increasing their proliferation in the presence of 0.5–3.0 mM of butyrate [10].

In our studies, the tested cancer cell lines exposed to butyrate showed differences in their proliferation rate. A similar observation was made by Zhang et al. [36], although the threshold of the proliferation-reducing butyrate concentration was higher in our study (1.5 mM and 5 mM for HCT116 and HT-29 after 48 h incubation, respectively, compared to 0.5 mM and 2 mM for HCT116 and HT-29 after 24 h incubation, respectively). However, the significant increase in the population of apoptotic cells detected by Zhang et al. [36], was at 1 mM of butyrate for both cell lines, which is congruent with our results.

The response of HCT116 and HT-29 cells to butyrate was to some extent similar regarding the cell cycle and apoptosis analyses (reduction of cells in S phase and an increase of those in G2/M phase), although it was more profound in the HCT116 cells (reduction of the cell pool in G0/G1 phase and a higher proportion of SUB G1 population, as well as a drastic increase in the population of late apoptotic/dead cells). Compared to tumorigenic cells, the NCM460D cells exposed to butyrate responded in a different way, i.e., an increased proliferation at low concentrations and inhibition at higher doses. Reduction of proliferation rate in 24 h cultures with high butyrate concentrations and in 48 h cultures might result from both the activity of butyrate as a donor of acetyl-CoA and apoptosis induction or from nutrient depletion after a very intensive cell growth. The impact of as low as 0.25 to 2 mM of butyrate on NCM460 cells was recently described and compared to HCT116 cells by Zeng et al. [37] and their findings are partially consistent with ours, i.e., butyrate induced apoptosis in higher rates in HCT116 cell than in NCM460; however, it did not increase the proliferation rate of the latter ones. It is of importance that Zeng et al. [37] conducted their experiments using DMEM medium for both cell lines, whereas in our study, the culture medium recommended by the NCM460D cells’ provider was used. Nevertheless, the observed effect of 15 h incubation with butyrate on expression of proteins involved in cell proliferation, in particular on the level of pERK1/2 (elevated in NCM460 and decreased in HCT116) is convergent with our results obtained in a 24 h experiment.

In our study, we investigated HCT116 and HT-29 cells which in studies by Chen et al. [20] were less sensitive to phytate than SW620 cell lines. We applied low and medium concentrations of PA (0.5–5 mM) and relatively short exposure time (24 h). In such conditions there was no significant inhibition of cell proliferation of HT-29 cells at all tested concentrations of PA, which is consistent with previous reports [19,20], whereas proliferation of HCT116 cells was inhibited by 1.0–5.0 mM PA. In studies by Liu et al. [38] phytate showed an inhibitory effect on HT-29 cells through modulation of the PI3K/Akt pathway, in particular, phytate at concentrations of 200–400 µg/mL decreased the expression of PI3K, Akt, and pAkt (the authors do not show the pAkt/Akt ratio which informs about the activation of the pathway), whereas phytate promoted its downstream signaling target, caspase-9. We could not confirm these findings; however, we revealed that PA decreased phosphorylation of ERK1/2 and expression of *iNOS*, which may contribute to the increased apoptosis [39] prolonged (three days) exposition of HT-29 cells to as high as 5 mM of InsP6 leads to G1 cell cycle arrest [40].

Despite the widely investigated effect of phytate, there is no data on its influence on the NCM460D cell line and, to the best of our knowledge, this is the first study describing the effect of phytate on the cell lines derived from healthy tissue. Compared to cancer cells, phytate did not affect the proliferation of NCM460D cells and cell cycle, although it increased the proportion of late apoptotic/dead cells, which was confirmed by the significantly decreased pAkt level. Only in NCM460D cells, phytate applied alone significantly increased pERK1/2 which is the effector protein in the extracellular signal-regulated kinase (ERK) mitogen-activated protein (MAP) kinase signaling pathway that regulates many important cellular processes including cell survival, cell differentiation, apoptosis, invasion, and inflammation. Activation of the ERK pathway is one of the key mechanisms for the initiation and progression in many human cancers including colorectal cancer [41], thus the decrease of pERK in HT-29 cells by both phytate and butyrate proves their anticancer activity. On the other hand, the recent reports explain the positive role of ERK pathway activation in regeneration of healthy small intestinal epithelium [42], which could take place in case NCM460D cells, as their proliferation decreased and the inflammatory markers (NFκB and *IL8*) were unchanged. Moreover, similar response (i.e., elevated pERK) of NCM460 cells to sulforaphane (isothiocyanate present in vegetables) was reported [43]. These findings suggest that activation of the ERK pathway in healthy colonocytes in response to natural substances of pro-apoptotic activity protects colonic epithelium from a loss of integrity and tightness.

Simultaneous exposition of cells to phytate and butyrate revealed that the HCT116 cell line was more sensitive to these factors than HT-29 cells, whereas changes observed in normal colonocytes were the weakest. The strongest effect of PA1B1 observed in HCT116 cells were on proinflammatory mediators (*TNFα* and *IL8*) and the related PI3K/Akt oncogenic signaling pathway. IL8 (CXCL8) participates in most phases of tumor development from cell proliferation and angiogenesis to cancer metastasis [44]. CXCL8 signaling can be induced by numerous factors associated with both neoplasia and inflammation [45]. TNFα is one of these factors and plays a critical role for maintaining the intestinal integrity and for the pathogenesis of intestinal inflammation; therefore, its functions can be both beneficial as well as harmful depending on the inflammatory context [46]. Furthermore, the CXCL8-activated signaling pathway is the PI3K/Akt pathway, which was activated in PA1B1-treated HCT116 cells [45]. Taking into account that HCT116 cells were strongly affected by PA1B1 treatment (the highest proportion of late apoptotic and dead cells (Figure 2), it can be suggested that the observed increase in pAkt was due to compensation mechanisms observed in viable cells. HCT116 cells can acquire a resistance to low concentrations of butyrate (up to 1.6 mM) [47] and this resistance is due to a switch from canonical to non-canonical Wnt signaling resulting in an increase of pAkt levels which represents a survival mechanism in mammalian cells exposed to pro-apoptotic stimuli [48]. Additionally, the increased expression of *IL8* in PA1B1-treated HCT116 cells may indicate a developing resistance [49].

Changes in the population size of apoptotic or necrotic cells were observed in all investigated cell lines. However PA1B1-treated HT-29 cells were undergoing early apoptosis, whereas in the NCM460D cell line both early apoptotic and late apoptosis/dead cell proportions increased but to much lower extent than in cancer cells. It should be stressed that in the applied experimental conditions, the pro-proliferative effect of butyrate on NCM460D cells was weakened in the presence of phytate. Similarly to cancer cells, NCM460D cells exposed to phytate and butyrate increased (but to a much lower extent) the late apoptotic and dead cells percentage and proportion of SUB G1 subpopulation, and decreased the percentage of viable cells. The activation of the ERK pathway was statistically insignificant but accompanied by a decreased *IL8* expression indicating the non-tumor-related activation of the ERK pathway [44,50]. It seems that the activation of the ERK pathway, lack of DNA synthesis inhibition together with constitutively high *iNOS* expression abolished the pro-apoptotic effect of the combined phytate and butyrate. Inducible nitric oxide synthase (iNOS) is expressed in many cell types, including epithelial cells, under both normal and pathological conditions [51]. Whether the relatively high *iNOS* expression in NCM460D cells determines their proliferation ability is a question that further studies should answer.

Synergistic effects of phytochemicals (also those originating from food and herbs) or combinations of phytochemicals and chemotherapeutic agents in vitro have been reported and, in most cases, their simultaneous application enhanced the anticancer activity [52]. Sparse studies have compared such effects in tumorigenic and non-tumorigenic colonocytes (such as the NCM460 cell line) reporting a lack of adverse effects of genitifib and decitabine on healthy cells [35]. Similarly, reports on simultaneous anticancer activity of diet-related substances that might be naturally and permanently present in the gut are scarce. In one such study, Drago et al. [53] showed that propolis augmented apoptosis of butyrate-sensitive colon cancer cells (HCT116 cell line) and re-sensitized butyrate-resistant colon cancer cells to apoptosis was possible by suppressing AKT signaling and downregulating the JAK/STAT pathway. The authors postulated that nutrient combinations that are a source of apoptosis inducers and inhibitors of compensatory cell proliferation pathways (e.g., AKT signaling) may produce high levels of programmed death in colon cancer cells. Unfortunately, they did not investigate that activity on healthy colonocytes.

## 5. Conclusions

The colonic epithelium is never exposed to a single factor and the studies on the effect of combinations of factors naturally present in the intestines provide important information on phenomena occurring on the border of the intestinal lumen and organism. The combined effect of butyrate and phytate was cell line-specific although some cancer cells-specific patterns of response can be indicated. The presence of both factors enhanced their pro-apoptotic effect in cancer cells, while in the healthy ones phytate suppressed the pro-proliferative action of butyrate and activated a pro-survival pathway. Results of this study give evidence on mechanisms by which dietary components might be involved in a maintenance of a healthy colonic epithelium. More studies in both in vivo and in vitro models are needed to explain modulatory mechanisms of this phenomenon and to confirm or deny its universality in healthy epithelial tissues. Based on the results obtained we postulate that studies on both cancer and/or non-cancer colonocytes should be carried out in the presence of butyrate as the permanent component of colonic contents. This should be of particular importance when anti-proliferative/pro-apoptotic activity of a compound is studied, or an inflammatory status of cells is investigated.

## Figures and Tables

**Table 1:** Composition of the phytate preparation determined with the HPLC-MS method.

Inositol Phosphates ^1^	Content	
	µg/mL	%
1-IP1	1.272	0.018
1,2-IP2	11.75	0.168
4,5-IP2	11.75	0.168
1,4,5-IP3	5.380	0.077
1,2,6-IP3	1.871	0.027
1,5,6-IP3	23.70	0.338
1,2,5,6-IP4	50.20	0.716
1,2,3,6-IP4	29.35	0.418
1,2,3,5,6-IP5	2525	36.01
IP6	4352	62.06
Total	7012	100

^1^ Refer to Table S1.

**Table 2:** The effect of 1 mM phytate and/or 1 mM butyrate on cellular processes in cancer and healthy human colonocytes.

Tested Factor	Cellular Processes ^1^	Cancer Colonocytes		Healthy Colonocytes
		HCT116	HT-29	NCM460D
1 mM phytate	Proliferation	↓	↔	↔
	Apoptosis/cell death	↑/↑	↔/↔	↔/↑
	Inflammation	↔	↔	↓
	Survival	↔	↓↓	↑
1 mM butyrate	Proliferation	↔	↔	↑↑
	Apoptosis/cell death	↑↑↑/↑↑	↑/↑	↑/↑
	Inflammation	↑	↔	↓
	Survival	↔	↓↓	↔
1 mM phytate + 1 mM butyrate	Proliferation	↓↓	↔	↔
	Apoptosis/cell death	↑↑/↑↑↑	↑↑↑/↑	↑↑/↑↑
	Inflammation	↑↑	↔	↓
	Survival	↔	↓↓	↑

^1^ Cellular processes were determined based on: proliferation—colorimetric test with Wst-1 reagent, expression of *PTEN* and *iNOS*; apoptosis/cell death—Annexin V detection, active caspase 3 levels, expression of *BCL2*, *BCLXL* genes; inflammation—expression of *NFκB1*, *TNFα*, *IL8*, genes and the level of NFκB; survival—pERK1/2 level (see Figure 1, Figure 2, Figure 3 and Figure 4 and Figures S1, S3 and S4) are included in the table. ↑—stimulation, ↓—inhibition or ↔—no effect; one, two, or three arrows—weak, moderate, or strong effect, respectively.

## Data Availability

Data presented in this study are available on request from the corresponding author.

## Supplementary Materials

The following are available online at https://www.mdpi.com/article/10.3390/nu13061887/s1, Figure S1: Proliferation rate (%) of HCT116, HT-29, and NCM460D cells in the presence of butyrate or phytate applied separately, Figure S2: Comparison of basal gene expression levels in investigated cell lines, Figure S3: Impact of 24 h incubation of cancer (HCT116 and HT-29) and healthy (NCM460D) colonocytes in the presence of 1 mM of phytate (PA1.0), 1 mM of butyrate (B1.0), or their combination (PA1B1) on levels of active Caspase 9 and cyclin A, Figure S4: Exemplary immunoblots showing the impact of 24 h incubation of cancer (HCT116 and HT-29) and healthy (NCM460D) colonocytes in the presence of 1 mM of phytate (PA1.0), 1 mM of butyrate (B1.0), or their combination (PA1B1) on levels of PTEN, p-Akt, Akt, NFκB, pERK1/2, ERK1/2, active caspase 3 and pro-caspase 3, Table S1: List of the authentic standards used for HPLC-MS analysis of the phytate preparation, Table S2: Primers used in the studies.
