# Supplementary material for: Phytate and Butyrate Differently Influence the Proliferation, Apoptosis and Survival Pathways in Human Cancer and Healthy Colonocytes
Source: Nutrients. 2021 May 31;13(6):1887. doi: 10.3390/nu13061887 (PMC8230256; doi:10.3390/nu13061887)
Supplement: Supplementary file 1 [file nutrients-13-01887-s001.zip › Supplementary Material Figure S3.pdf]

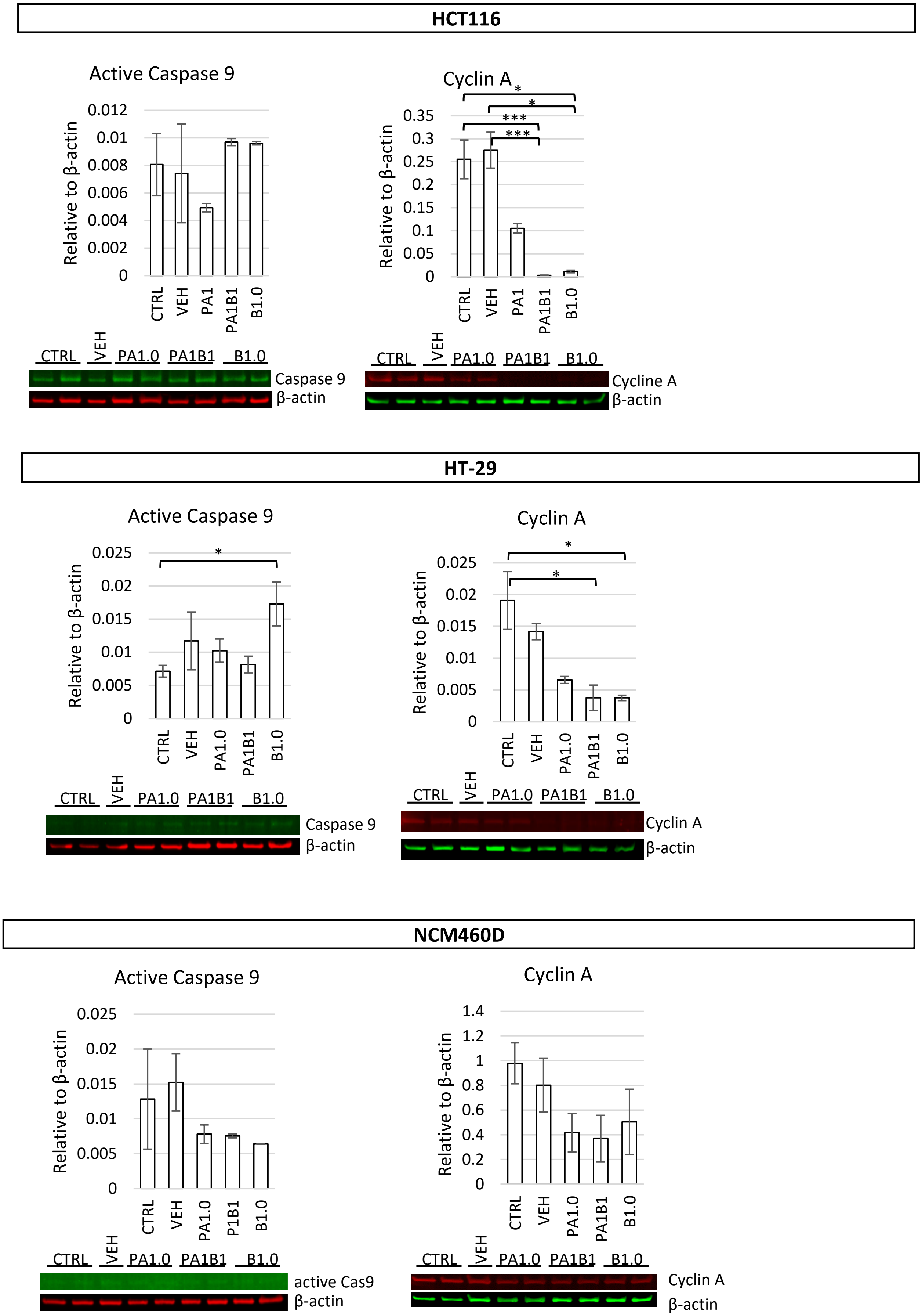

Figure S3. Impact of 24h-incubation of cancer (HCT116 and HT-29) and healthy (NCM460D) colonocytes in the presence of 1 mM of phytate (PA1.0), 1 mM of butyrate (B1.0) or their combination (PA1B1) on levels of active Caspase 9 and cyclin A. CTRL – control cells incubated without supplements, VEH – cells incubated in the presence of PBS.
