# Supplementary material for: Phytate and Butyrate Differently Influence the Proliferation, Apoptosis and Survival Pathways in Human Cancer and Healthy Colonocytes
Source: Nutrients. 2021 May 31;13(6):1887. doi: 10.3390/nu13061887 (PMC8230256; doi:10.3390/nu13061887)
Supplement: Supplementary file 1 [file nutrients-13-01887-s001.zip › Supplementary Material Figure S4.pdf]

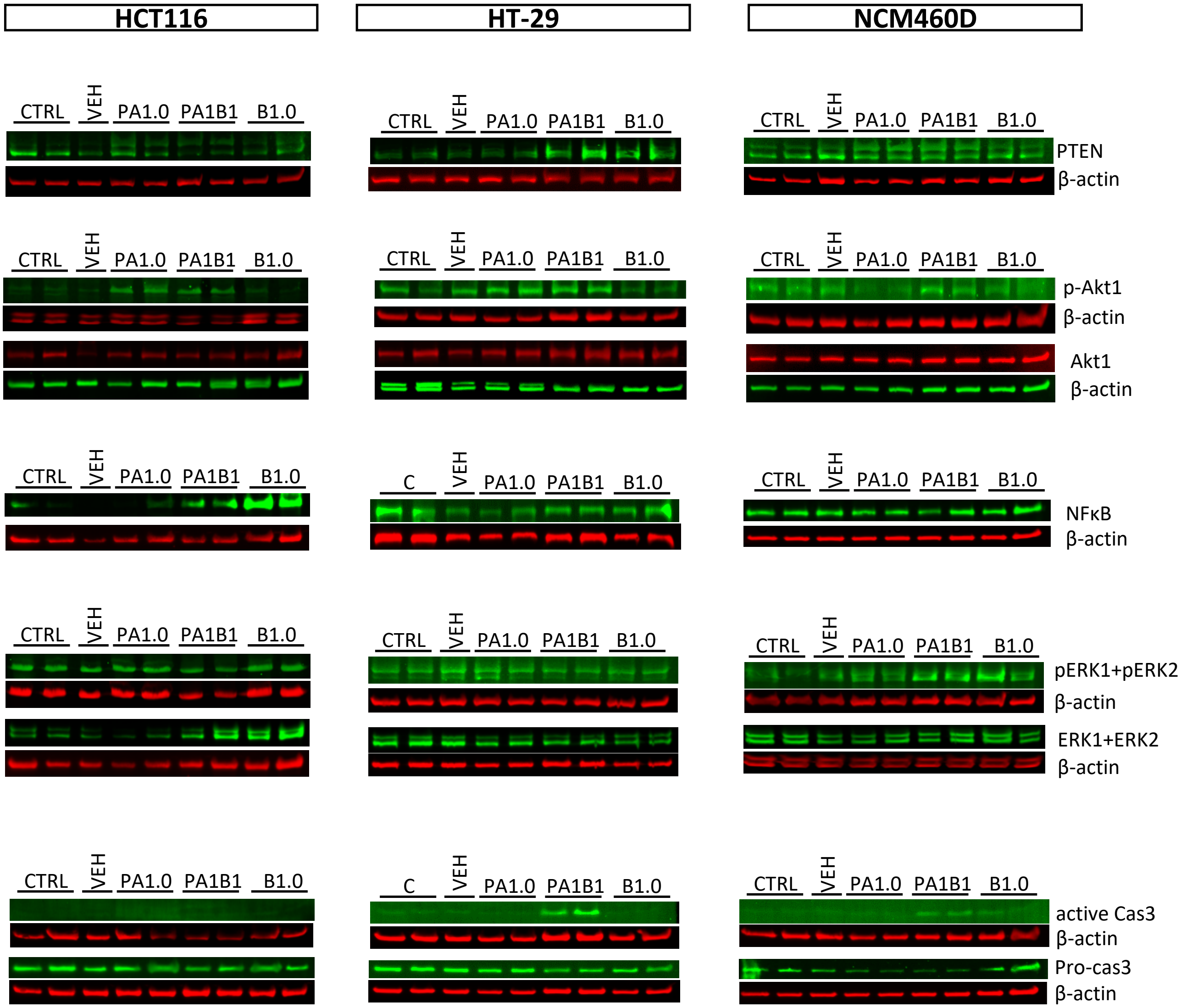

Figure S4. Exemplary immunoblots showing the impact of 24h-incubation of cancer (HCT116 and HT-29) and healthy (NCM460D) colonocytes in the presence of 1 mM of phytate (PA1.0), 1 mM of butyrate (B1.0) or their combination (PA1B1) on levels of PTEN, p-Akt, Akt, NFκB, pERK1/2, ERK1/2, active caspase 3 and pro-caspase 3. CTRL – control cells incubated without supplements, VEH – cells incubated in the presence of PBS.
