# Supplementary material for: Phytate and Butyrate Differently Influence the Proliferation, Apoptosis and Survival Pathways in Human Cancer and Healthy Colonocytes
Source: Nutrients. 2021 May 31;13(6):1887. doi: 10.3390/nu13061887 (PMC8230256; doi:10.3390/nu13061887)
Supplement: Supplementary file 1 [file nutrients-13-01887-s001.zip › Supplementary Material Table S1.pdf]

Supplementary Information

Table S1. List of the authentic standards used for HPLC-MS analysis of the phytate preparation

	Name of a standard	Producer, catalog number	Inositol phosphate
1.	D-*myo*-Inositol 1,2,3,5,6-pentakisphosphate (decasodium salt)	Sigma, 76667-1MG	1,2,3,5,6-IP5
2.	D-*myo*-Inositol-1,2,3,6-tetrakisphosphate (tetrapotassium salt)	Cayman Chemical, 10008450	1,2,3,6-IP4
3.	D-*myo*-Inositol-1,2,5,6-tetrakisphosphate (ammonium salt)	Santa Cruz Biotechnology, sc-214815	1,2,5,6-IP4
4.	D-*myo*-Inositol-1,2,6-triphosphate (sodium salt)	Santa Cruz Biotechnology, sc-223918	1,2,6-IP3
5.	D-*myo*-Inositol 1,4,5-trisphosphate (hexapotassium salt)	ENZO Life Sciences, ALX-307-009-M001	1,4,5-IP3
6.	D-*myo*-Inositol-1,5,6-triphosphate (sodium salt)	Santa Cruz Biotechnology, sc-205286	1,5,6-IP3
7.	D-*myo*-Inositol-1,2-diphosphate (sodium salt)	Santa Cruz Biotechnology, sc-223910	1,2-IP2
8.	D-*myo*-Inositol 1,4-bisphosphate (potassium salt)	Santa Cruz Biotechnology, sc-214818	1,4-IP2
9.	D-*myo*-Inositol-4,5-diphosphate (sodium salt)	Santa Cruz Biotechnology, sc-221514	4,5-IP2
10.	D-*myo*-Inositol-1-phosphate (sodium salt)	Santa Cruz Biotechnology, sc-223909	1-IP1
11.	*myo*-Inositol	Santa Cruz Biotechnology, 202714	IP
