# Supplementary material for: Phytate and Butyrate Differently Influence the Proliferation, Apoptosis and Survival Pathways in Human Cancer and Healthy Colonocytes
Source: Nutrients. 2021 May 31;13(6):1887. doi: 10.3390/nu13061887 (PMC8230256; doi:10.3390/nu13061887)
Supplement: Supplementary file 1 [file nutrients-13-01887-s001.zip › Supplementary Material Table S2.pdf]

**Supplementary Information**

Table S2. Primers used in the studies

| Name | Sequence (5' → 3') | $T_a$ (°C)[1] | Target | Reference |
|---|---|---|---|---|
| PTEN-f<br>PTEN-r | CCAGTGGCACTGTTGTTTCACA<br>CAGGTAACGGCTGAGGGAGCTC | 60 | Phosphatase and tensin homolog | Larson Gedman et al. 2009 |
| hBcl-xl-F<br>hBcl-xl-R | GATCCCCATGGCAGCAGTAAAGCAAG<br>CCCCATCCCGGAAGAGTTCATTCACT | 63 | B-cell leukemia/ lymphoma X long isoform | Larson Gedman et al. 2009 |
| hBcl2-F<br>hBcl2-R | CTGCACCTGACGCCCTTCACC<br>CACATGACCCCACCGAACTCAAAGA | 61 | B-cell leukemia/ lymphoma 2 | Larson Gedman et al. 2009 |
| iNOS-f<br>iNOS-r | ACAACAAATTCAGGTACGCTGTG<br>TCTGATCAATGTCATGAGCAAAGG | 58 | Inducible nitric oxide synthase | De Boo and Kopecka et al. 2009 |
| NFκB-f<br>NFκB-r | ACAAATGGGCTACACCGAAG<br>TAGGGCTTTGGTTTACACGG | 55 | Nuclear factor, light chain/polypeptide, p105 | Banno et al. 2004 |
| TNF-R-f<br>TNF-R-r | CACCAGGTGTGATTCAGGTG<br>CCCCACTGTGCTTTGTACCT | 54 | Tumor necrosis factor receptor superfamily member 10b (TNFRSF10B) | Hofer et al. 2013 |
| IL-8-f<br>IL-8-r | ACATACTCCAAACCTTTCCACCC<br>CAACCCTCTGCACCCAGTTTTC | 58 | Interleukin 8 | Li et al. 2001 |
| ACTB-f<br>ACTB-r | TGAAGTGTGACGTGGACATC<br>ACTCGTCATACTCCTGCTTG | 60 | β-actin | Galligan et al. 2009 |

[1] Ta – annealing temperature

Literature


Larson Gedman A, Chen Q, Kugel Desmoulin S, Ge Y, LaFiura K, Haska CL, Cherian C, Devidas M, Linda SB, Taub JW, Matherly LH. The impact of NOTCH1, FBW7 and PTEN mutations on prognosis and downstream signaling in pediatric T- cell acute lymphoblastic leukemia: A report from the Children's Oncology Group. Leukemia. 2009, 23(8), 1417–1425.

De Boo S, Kopecka J, Brusa D, Gazzano E, Matera L, Ghigo D, Bosia A, Riganti C. iNOS activity is necessary for the cytotoxic and immunogenic effects of doxorubicin in human colon cancer cells. Molecular Cancer 2009, 8,108.

Banno T., Gazel A., Blumenberg M. Effects of Tumor Necrosis Factor-α (TNFα) in epidermal keratinocytes revealed using global transcriptional profiling. The Journal Of Biological Chemistry, 2004, 279(31), 32633–32642.

Hofer D., Schwach G., Ghaffari Tabrizi-Wizsy N., Sadjak A., Sturm S., Stuppner H., Pfragner R. Christia vespertilionis plant extracts as novel antiproliferative agent against human neuroendocrine tumor cells. Oncology Reports, 2013, 29, 2219-2226. https://doi.org/10.3892/or.2013.2367

Li A., Varney M.L., Singh R.K. Expression of interleukin 8 and its receptors in human colon carcinoma cells with different metastatic potentials. Clinical Cancer Research, 2001, 7, 3298–3304.

Galligan C.L., Matsuyama W., Matsukawa A., Mizuta H., D.R. Hodge D.R., Howard O.M.Z., Yoshimura T. Up-regulated expression and activation of the orphan chemokine receptor, CCRL2, in rheumatoid arthritis. Arthritis & Rheumatism, 2004, 50(6), 1806–1814. DOI 10.1002/art.20275.
