# Supplementary figures and images for: Phytate and Butyrate Differently Influence the Proliferation, Apoptosis and Survival Pathways in Human Cancer and Healthy Colonocytes
Source: Nutrients. 2021 May 31;13(6):1887. doi: 10.3390/nu13061887 (PMC8230256; doi:10.3390/nu13061887)

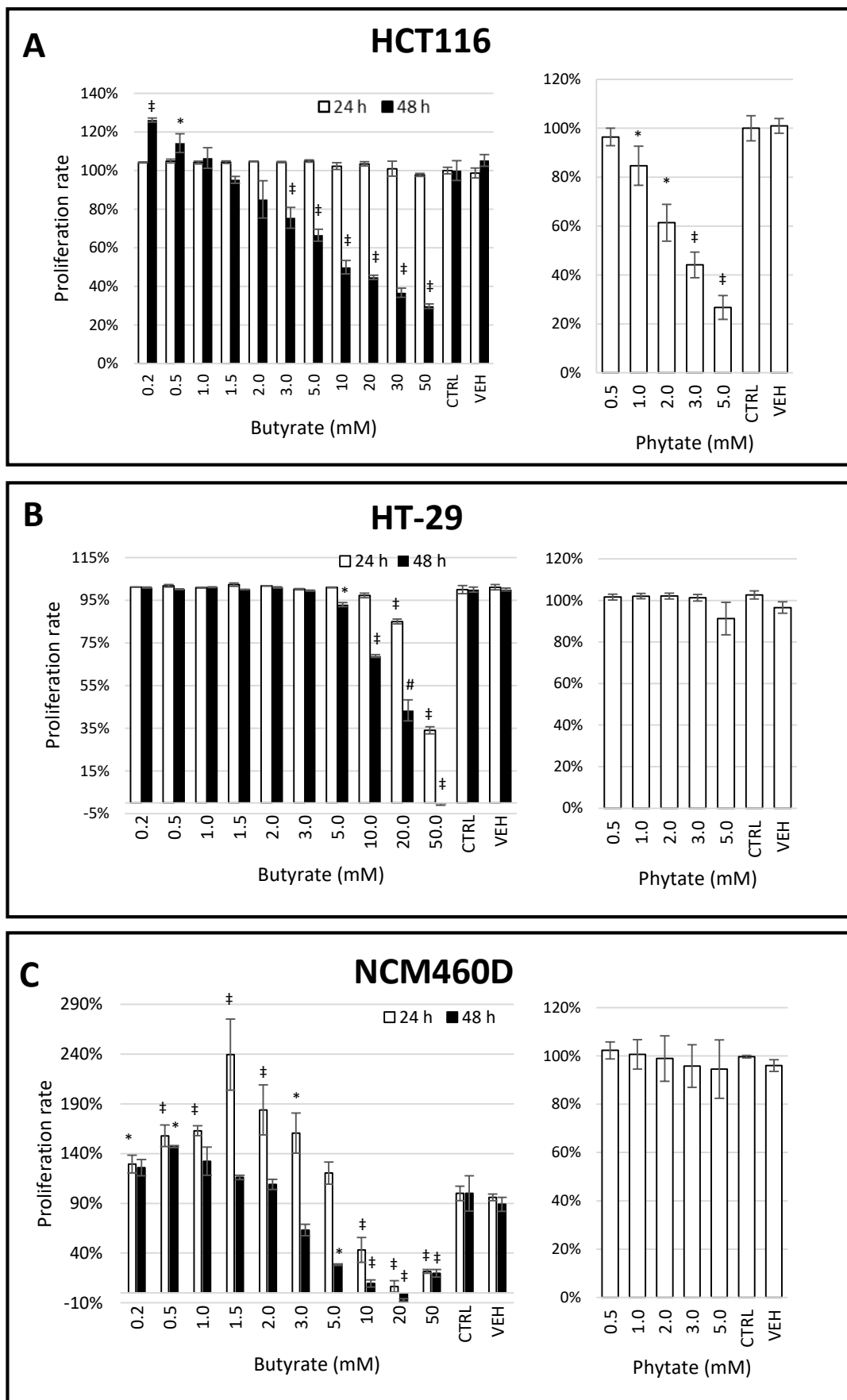

Supplement: Supplementary file 1 [file nutrients-13-01887-s001.zip › Supplementary Material Figure S1.pdf]
